# Training in Implementation Practice Leadership (TRIPLE): evaluation of a novel practice change strategy in behavioral health organizations

**DOI:** 10.1186/s13012-019-0906-2

**Published:** 2019-06-20

**Authors:** Enola Proctor, Alex T. Ramsey, Matthew T. Brown, Sara Malone, Cole Hooley, Virginia McKay

**Affiliations:** 1 Brown School, Washington University in St. Louis, 1 Brookings Drive, St. Louis, MO 63130 USA; 2 Department of Psychiatry, Washington University School of Medicine, 660 S Euclid Ave, St. Louis, MO 63110 USA; 3 Institute for Public Health, Washington University in St. Louis, 660 S Euclid Ave, St. Louis, MO 63110 USA

**Keywords:** Training, Implementation, Practice, Leadership, Evaluation, Practice change, Behavioral health

## Abstract

**Background:**

Effective leadership for organizational change is critical to the implementation of evidence-based practices (EBPs). As organizational leaders in behavioral health organizations often are promoted from within the agency for their long-standing, effective work as counselors, they may lack formal training in leadership, management, or practice change. This study assesses a novel implementation leadership training designed to promote leadership skills and successful organizational change specific to EBP implementation.

**Methods:**

We conducted a pre-post outcome evaluation of the Training in Implementation Practice Leadership (TRIPLE), delivered via three in-person, half-day training sessions, with interim coaching and technical support. Sixteen mid-level leaders (75% female, 94% Caucasian, mean age 37 years) from 8 substance abuse treatment agencies participated. Professional roles included clinical managers, quality improvement coordinators, and program directors. Participants completed surveys prior to the first and following the final session. At both time points, measures included the Implementation Leadership Scale, Implementation Climate Scale, and Organizational Readiness for Implementing Change Scale. At post-test, we added the Training Acceptability and Appropriateness Scale (TAAS), assessing participant satisfaction with the training. Qualitative interviews were conducted 6 to 8 months after the training.

**Results:**

Most participants (86% and 79%, respectively) reported increased implementation leadership skills and implementation climate; paired samples *t* tests indicated these pre-post increases were statistically significant. Implementation leadership scores improved most markedly on the Proactive and Knowledgeable subscales. For implementation climate, participants reported the greatest increases in educational support and recognition for using EBP. Post-test scores on the TAAS also indicated that participants found the training program to be highly acceptable and appropriate for their needs. Qualitative results supported positive outcomes of training that resulted in both increased organizational implementation as well as leadership skills of participants.

**Conclusions:**

This training program represents an innovative, effective, and well-received implementation strategy for emerging behavioral healthcare leaders seeking to adopt or improve the delivery of EBPs. Reported implementation leadership skills and implementation climate improved following the training program, suggesting that TRIPLE may have helped fulfill a critical need for emerging behavioral healthcare leaders.

**Electronic supplementary material:**

The online version of this article (10.1186/s13012-019-0906-2) contains supplementary material, which is available to authorized users.

## Background

Effective leadership and organizational change are critical to the implementation of evidence-based practices (EBPs). Leaders foster an organization’s climate and employees’ attitudes toward EBP implementation [1], support innovation and implementation efforts by securing funding, allocating resources, and enforcing policies [2], and directly impact the success of efforts to implement practice change [3, 4]. The implementation of practice change involves many different stakeholders or “actors” [5] including agency directors, clinical managers, clinical supervisors, and direct providers. In behavioral health organizations, responsibility for introducing and ensuring high-quality delivery of EBPs often falls on clinical supervisors or middle-level organizational leaders. Because agencies often promote leaders from within clinics for their long-standing and effective work as front-line providers, many new supervisors and managers lack formal training in leadership or practice change [6].

Yet, the success of practice change requires leadership for a strong implementation climate in which high-quality care by new direct service providers is expected, rewarded, and supported. Prior research has highlighted two types of programs in particular—substance use disorder treatment programs delivering motivational interviewing and mental health clinics delivering interventions for post-traumatic stress disorder—in which evidence-based programs require effective leadership for implementation [7, 8]. Although a growing number of programs offer training in implementation science, training for implementation practice is scarce [9, 10], as is reported evaluation of such training. As imminent drivers of organizational change, emerging behavioral healthcare leaders must be engaged in well-designed training programs to foster the competencies, decision-making, and management skills that comprise effective implementation practice leadership.

To address this gap, our team developed, delivered, and evaluated a training program for clinical leaders and managers, the Training in Implementation Practice Leadership, or TRIPLE. We designed TRIPLE to promote leadership skills and successful organizational change in regard to EBP implementation. This paper reports the content, format, and results of a mixed-method evaluation. The evaluation addressed three broad questions:

1. How receptive are practice leaders to TRIPLE?

2. Does the training improve leaders’ knowledge and behavior related to EBP?

3. Does the training improve implementation of EBP within organizations?

## Methods

### TRIPLE

Our evaluation of the TRIPLE program used a mixed-methods, pre-test/post-test, single-group design. We assessed change in knowledge and skills of trainees through pre-post implementation research measures (scales). Our mixed-methods approach entailed a Quant➔Qual explanatory sequential design [11] such that we conducted the quantitative surveys at the beginning and end of the class, followed with qualitative interviews approximately 6 months after the training for enhanced understanding of training impact.

### Training rationale

The Center for Dissemination and Implementation (CDI) of the Institute for Public Health (IPH) at Washington University in St. Louis offered the training. Leaders of the practice community expressed the desire for support in implementing EBPs. Several factors had stimulated this interest including: the social work school’s long-established EBP curriculum for master’s students [12], a seminar series in Community Academic Partnership on Addiction (CAPA) dissemination and implementation offered by the IPH CDI [13], and the social work school’s CAPA program (CAPA) [14]. The CAPA program https://addiction-partnership.wustl.edu/ is designed to establish strong connections with organizations throughout the St. Louis and the surrounding region that provide services related to the problems of addiction. Its mission is to train current and future workers, create teaching-learning opportunities, and conduct collaborative research that improves systems of addiction services using a partnership approach. CAPA leaders are on the distribution list for CDI seminars and were periodically invited to consultation and networking sessions with guest speakers. Because several agency leaders requested additional support, CDI leaders decided to organize and offer TRIPLE.

### TRIPLE aims

This training program aimed to build participants’ skills to (1) assess the quality of current service delivery; (2) identify appropriate and feasible evidence-based policies, programs, and practices that address agency priorities; (3) persuasively introduce new EBPs and develop internal support for their use; (4) assess and increase stakeholder engagement in practice improvement; and (5) identify and use appropriate data to monitor the quality of their delivery and lead ongoing practice change.

### Invitation and recruitment

The CDI center director and manager (EP, MTB) sent a letter to directors and Chief Executive Officers (CEO)s of CAPA-affiliated agencies describing the training opportunity and invited them to nominate two or three clinical leaders and supervisors of behavioral health programs in their agencies to participate in the program (see Additional file 1). The letter described the training content, structure, requirements, and benefits.

### Participants

Invitations were sent to 8 agencies; all 8 agencies nominated clinical leaders for the TRIPLE program. Training participants included 16 mid-level leaders (75% female, 94% Caucasian, mean age 37 years), their demographics, and the demographics of the sets of participants who participated in the quantitative and qualitative portions of the survey are in Table 1. Professional roles included clinical managers, quality improvement coordinators, and program directors. The Washington University in St. Louis Institutional Review Board approved all evaluation protocols. We did not pay participants for participation.

**Table 1:** Participant demographics

	All	Quantitative	Qualitative
Participants	16	12	9
Agencies	8	7	5
% Female	75%	67%	78%
% Caucasian	94%	92%	89%
Mean age	37	35	41
Professional roles	Clinical managers, quality improvement coordinators, and program directors	Clinical managers, quality improvement coordinator, program directors, Associate CEO, addiction counselor	Clinical managers and program directors

### Training format

The CDI delivered the training in three half-day sessions, scheduled approximately 4 weeks apart from January to March 2017. The training format included lectures, individual and small group exercises, and reading assignments. Trainees developed and trialed a small-scale implementation project in the participant’s clinic setting. Participants shared contact information in an effort to promote ongoing networking. Experts in behavioral health implementation led the sessions, which included exercises to enhance participant skills required to drive the implementation of EBPs. Between sessions, the training offered participants optional conference calls with the experts for more in-depth coaching and technical support.

### Training content

Content focused on the knowledge, skills, and tools necessary to lead the implementation and evaluation of appropriate EBPs in participants’ respective agencies or clinics, as informed by research on training needs and D&I competencies [15, 16]. The registration fee included a textbook, *Improving Patient Care: The Implementation of Change in Clinical Practice* [17] for all participants. Although this book focuses heavily on medical care, its general content is appropriate for behavioral health clinics.

Table 2 outlines the curriculum. Training included content about models and frameworks for change (e.g., the implementation process); registries and web sources of evidence-based practices; strategies to overcome provider, organizational, client, and policy barriers to implement new practices; and approaches for quality assessment and continuous data monitoring to evaluate change. This training differed from broader leadership development opportunities in that it focused specifically on knowledge, competencies, and leadership abilities for implementing and evaluating evidence-based programs.

**Table 2:** Curriculum at a glance

Session 1—setting the stage/planning and engaging in change	Session 2—making it happen/executing the change	Session 3—keep improving/evaluating and reflecting on the change
Who is involved in practice improvement	Review of work since session 1 and 3 shared experiences from your site.	Principles of a learning organization *Reinforcing exercise*: quality measures
Organizational factors in the implementation of EBPs	Theories, models, and frameworks in EBP *Reinforcing exercise*: assessing practice properties	Break-out sessions reporting on successes and challenges
Key steps in implementation *Reinforcing exercise*: quality gap estimation	Leadership for change *Reinforcing exercise*: Stakeholders for change	Economics/cost evaluation
Team activity and reporting *Reinforcing exercise*: Implementation success stories	Strategies for implementing change *Reinforcing exercise*: agency motivators for implementing innovations	Team activity and reporting *Reinforcing exercise*: implementation of climate development plan
Discuss plans for small-scale implementation rialing and interim coaching *Reinforcing exercise*: sticky messages to facilitate change	Team activity and reporting	Quality improvement and performance management *Reinforcing exercise*: quality of care and EB practice
		Post-training evaluations and ‘What is next’

### Quantitative data collection

#### Data collection and measures

At the start of the first session and end of the last session, participants completed the following paper-based surveys: Implementation Leadership Scale (ILS), Implementation Climate Scale (ICS), and Organizational Readiness for Implementing Change (ORIC) Scale. At post-test, we added the Training Acceptability and Appropriateness Scale (TAAS) to assess participant satisfaction with the training. We also documented attendance for all participants.

#### Implementation Leadership Scale

The ILS (Implementation Leadership Scale) is a 12-item measure of the extent to which an individual leader identifies themselves as proactive, knowledgeable, supportive, and perseverant about the implementation of EBPs. All items were rated along a 0 (not at all) to 4 (very great extent) scale, with higher scores indicating more positive implementation leadership. The ILS has demonstrated very high internal consistency in the overall scale (α = .98), among the four subscales which include proactive, knowledgeable, supportive, and perseverant (α = .95–.96), as well as strong convergent and discriminant validity [18, 19]. The ILS has been used in a wide range of settings, including mental health agencies, child welfare, substance use disorder treatment centers, and hospitals.

#### Implementation Climate Scale

The ICS (Implementation Climate Scale) is an 18-item measure assessing the extent to which implementation of EBPs are expected, rewarded, and supported in an organization. All items were rated along a 0 (not at all) to 4 (very great extent) scale, with higher scores indicating more positive implementation climate. The ICS has demonstrated high-internal consistency in the overall scale (α = .91) and among the following six subscales: selection for openness (α = 0.91), recognition for EBP (α = 0.88), selection for EBP (α = 0.89), focus on EBP (α = 0.91), educational support for EBP (α = 0.84), rewards for EBP (α = 0.81), and strong construct validity [20].

#### Organizational readiness for implementing change scale

The ORIC is a 12-item measure assessing the behavioral and psychological preparedness of a collective organization to successfully implement new policies, programs, and practices. Psychometric evaluation reflected high-internal consistency for both the change commitment (α = .91–.92) and change efficacy (α = .88–.89) scales, as well as good validity [21]. All items were rated along a 0 (disagree) to 4 (agree) scale, with higher scores indicating more positive organizational readiness for implementing change.

#### Training Acceptability and Appropriateness Scale

The TAAS (Training Acceptability and Appropriateness Scale) is a 14-item measure to assess participants’ perceptions of the extent to which a training program was acceptable, feasible, and appropriate for their needs. The TAAS is an unpublished measure developed by Aaron Lyon that was found in the SIRC instrument review toolkit. The instrument was used for descriptive analyses. A reliability analysis of the data on the scale showed high internal consistency (Cronbach’s alpha = 0.95). All items were rated along a 0 (not at all) to 4 (extremely) scale, with higher scores indicating more positive perceptions about the training program. We used this measure at post-test only.

#### Data analysis and management

Once collected, the evaluation team entered, managed, and analyzed the data in Stata.

### Missing data

While 16 individuals participated in the workshops (*n* = 16), the number of usable pre-/post-test scale responses varied due to degree of completeness (ILS *n* = 13, ICS *n* = 12, ORIC n = 12). Two participants did not attend the last session to complete the post-test scales, and two other respondents had missing values in either the pre- or post-test scales. We conducted a series of chi-square tests and *t* tests to assess the demographic differences between the respondents with incomplete responses and the respondents with complete responses. We created a dichotomous variable indicating whether or not the respondent had any missing values (no = 0, yes =1). We ran chi-square tests with the missing values variable and the categorical demographic variables gender, race, level of education, licensure, and agency role. We ran a two-sample *t* test grouping the respondents by the missing values variable for the two continuous demographic variables: age and years of employment. These demographic comparison assessments yielded no significant results, suggesting that the respondents with missing values did not differ significantly from the other respondents.

We ran a series of two sample *t* tests grouping the respondents by the missing values variable with the continuous pre-test sum scores, post-test sum scores, and difference between pre-/post-test scores. There were no between-group differences for the pre-test scores. We excluded from the analysis the scores of the two participants who did not complete the post-test. The ORIC post-sum, the ICS difference score, and the ORIC difference score violated the equal variance assumption. For those two-sample group *t* tests, we accounted for the variance issue using Stata’s unequal variance calculation. We found no significant differences for the post and difference between pre-/post-test scores. These results suggest that the respondents with missing values did not differ significantly from those with complete responses in the outcome variables of interest. As such, we only included respondents who had complete pre- and post-test for the given scales in our analysis.

We conducted paired samples *t* tests in Stata v.14 to assess pre-test to post-test changes in the sum score and the subscale sum scores for the ILS, ICS, and ORIC. We also used descriptive analyses to examine all variables, including the TAAS. We used a two-tailed hypothesis test with a pre-determined *p* value cutoff of .05. We checked assumptions for the test; the outcome variable is continuous, we used matched pairs, there were no outliers, observations are independent, the difference between pre- and post-test were normally distributed, and the variance between pre- and post-test were not statistically different. We calculated effect sizes for the results using the function “esizei” in Stata.

### Qualitative methods

#### Participants and recruitment

Six to 8 months after the final training session, we reached out to each of the 16 training participants via email and phone for qualitative interviews. Nine individuals completed interviews, one declined, and six did not respond to the request.

#### Data collection and measures

We conducted interviews using a semi-structured interview guide (see Additional file 2). We arranged times to conduct the interview via telephone call at the participant’s convenience. We sent a copy of the informed consent sheet with the first email requesting the qualitative interview. Interviews lasted approximately 20–30 min and were audio recorded while the interviewer took notes.

The evaluation team developed the interview guide by pilot testing it with individuals who did not participate in the training (i.e., researchers at the institution). Two interviewers received training to conduct the interviews. They asked interviewees about their experiences leading up to the training, during the training, and in their organizations since the training.

#### Data analysis and management

Interviews were transcribed using rev.com verbatim. The research team de-identified the transcripts, reviewed them for accuracy, and destroyed audio recordings once the process was complete. Initially, the team created a codebook using the Kirkpatrick model [22] for evaluating training courses and iteratively adjusted it as appropriate to reflect emergent phenomena in the text. The framework evaluates training along four dimensions: (1) reaction, (2) learning, (3) behavior, and (4) results [23]. While we maintained each of these four board dimensions, the coding process revealed several sub-dimensions within the “results” domain (see “Results” section below). Table 3 describes each of the dimensions, sub-dimensions, and their application to the qualitative data.

**Table 3:** Qualitative codebook of evaluation dimensions

Dimension	Original description	Sub-category	Application to TRIPLE	Relevant quantitative data
Reaction	How participants feel about the training			78.6% of respondents rated high levels of acceptability and appropriateness
		Receptivity to the training	Participants reactions to the training prior to attending	
		Positive about the training	Positive comments participants made about the training.	
		Negative or neutral about the training	Negative or neutral comments participants made about the training.	
Learning	Increase in participant knowledge		Participant comments regarding things they learned in the training, or how the training changed their way of thinking.	Significant increase in self-competence (knowledge subscale of ILS), with large effect size
Behavior	Application of knowledge in job		Participant reports of applying their knowledge in their agency or changing how they approach their job based on what they learned in the training.	Significant increase in resilient behaviors (perseverant subscale of ILS), with large effect size
Results	Effect on the business or environment			Significant increases in supporting, rewarding, and valuing expertise in EBP implementation (educational support, recognition, and selection subscales of ICS), with medium to large effect sizes
		Changes in services delivery or practice	Attempting to implement or successfully implementing new EBPs/considering the evidence-base for current practices and making adjustments to achieve fidelity.	
		Changes in agency culture or climate regarding EBP	Changes in how other administrative staff view EBP or implement services. Also, includes enhanced collaboration among staff.	
		Improved staff knowledge/training	Changes made to help frontline staff learn more about EBPs, or understand why the agency uses EBPs.	
		Changes in evaluative approaches	Changes in data collection forms and how evaluation is approached within the agency.	

Once the team finalized the codebook (Appendix), two team members independently applied the codes to all transcripts. Codes were mutually exclusive. The two team members then reviewed coding and resolved any discrepancies to ensure consistent application of codes. They generated Analytic memos throughout the analysis process to document changes in the codebook, emergent codes, and initial interpretation of results.

Qualitative results are presented in conjunction with the quantitative analyses according to our research questions. We provide representative quotes to illustrate the variety of ways the training influenced participants and the agencies where they work.

## Results

### Receptivity to training

In response to our first research question, both quantitative and qualitative data reflect the receptivity of participants to TRIPLE, with the qualitative data contributing to a deeper understanding of this receptivity. Post-test sum scores on the TAAS (*M* = 45.7; SD = 8.1) indicated that 78.6% of the respondents rated the training between “quite a bit” and “extremely” acceptable and appropriate.

We also used attendance as one indication of training receptivity. All 16 participants attended session 1; 13 attended session 2; 14 attended session 3. Participation in the follow-up coaching calls was limited. Only four individuals called in and only two raised questions or comments.

The qualitative interviews indicated general positivity before the training as well as remaining positive about their attendance after completing the training. Many participants described excitement about the opportunity to engage in practice-related training. They were positive about how the training was presented and their overall experience within the training program. However, some interviewees indicated neutrality or negativity toward attendance. Some of these individuals described that participation was still useful, while some stated that the training did not align with their perceived current needs within the organization. Participants also described different ways of learning about the training. While some heard about it on their own, others were nominated by their bosses or encouraged to participate. Those who were encouraged to go by another leader described mixed feelings about that: some felt honored that they were asked, while others found it to be another commitment that they did not have time to participate in.

### Training and improvements in leadership knowledge and behavior

For our second research question, we observed a convergence of quantitative and qualitative data, with the qualitative data enriching the favorable quantitative data. Participants’ scores on the ILS increased significantly from pre-test to post-test (mean difference = 6.0, 95% CI, 1.3–10.7) (see Table 3). This improvement corresponds with a pre-post effect size of *d* = .93 (large). The majority (85%) of participants reported an increase in their score. The knowledge subscale (mean difference = 1.8, 95% CI, .6–3.1) and the perseverant subscale (mean difference = 1.8 95% CI, .5–3.2) increased by a statistically significant degree, representing pre-post effect sizes of *d* = .85 (large) for knowledge and *d* = 1.08 (large) for perseverant.

The qualitative results highlighted numerous areas of learning and self-identified individual behavior change as well as individual skill development. Individuals broadly described their learning about implementation and steps to implementation, often focusing on the need for specific stakeholder engagement. For example, one participant stated, “It helped me to think more deeply…and to plan for some of the barriers and some of the things that get in the way of successfully implementing a practice.” Others were able to describe learning that translated into behavior changes, such as the way they communicate with or provide education to their employees.

### Training and improvements at the organization level

ICS scores increased significantly from pre-test to post-test, (mean difference = 6.7, 95% CI, 1.5–11.9) (see Table 3). This increase corresponds with an effect size of *d* = .72 (medium to large). The majority (75%) of participants reported an increase in their score. The educational support (mean difference = 1.6, 95% CI, .6–2.6), recognition (mean difference = 1.7, 95% CI, .1–3.3), and selection subscales (mean difference = 1.9, 95% CI, .5–3.3) increased by a statistically significant degree, representing pre-post effect sizes of *d* = .77 (medium to large) for educational support, *d* = .92 (large) for recognition, and *d* = .63 (medium) for selection.

Organizational readiness for implementing change scale scores trended upward from pre-test to post-test; however, this was not a statistically significant increase (mean difference = 2.9, 95% CI, − 2.3-8.2) (see Table 4). More than half (58%) of participants reported an increase in their score from pre-test to post-test.

**Table 4:** Pre-test/post-test change in the mean of sum scores (SD) for key outcomes

	ILS *M* (SD)	ICS *M* (SD)	ORIC *M* (SD)	TAAS *M* (SD)
Pre-test	31.31 (7.94)	40.00 (10.37)	33.83 (9.09)	
Post-test	37.31 (4.44)	46.67 (8.00)	36.75 (5.28)	45.71 (8.10)
Mean diff (95%CI)	6.00 (1.31–10.69)	6.67 (1.46–11.87)	2.92 (2.32–8.15)	
*p* value	0.0165	0.0167	0.2457	

Note: all items are based on a sum score. The total points possible for each scale: ILS = 48 points, ICS = 72 points, ORIC = 48 points, and TAAS = 56

*ILS* Implementation Leadership Scale

*ICS* Implementation Climate Scale

*ORIC* Organizational Readiness for Implementing Change scale

*TAAS* Training Acceptability and Appropriateness Scale

Most of the organizational-level changes reported in interviews pertained to training and education. Participants were able to articulate different approaches to communication within teams as well as being more intentional and training people before an organizational change rather than hastily rolling out a new initiative. Participants often specifically mentioned communication prior to trainings or rolling out new innovations. One respondent, although hesitant to attend the training initially, described the broad applicability and flexibility of the techniques learned. While not rolling out the intended EBP, that individual reported being able to utilize skills taught in the training in a variety of situations stating, “As I was training people, there were some people that didn’t really think [the new practice] was a good idea…it has changed how I approach some people in training, to work more not so much on the ins and outs, but why we’re doing it.”

While some participants discussed future plans regarding restructuring or financial incentives, no organizations had implemented these structural changes at this point. However, several organizations were re-examining their existing services as a result of the training. Individuals also described secondary changes within the agency from participating in the training. They linked the training to changes within the culture of the agency, including more buy-in and a “heightened awareness of how we’re approaching and how we’re implementing things.” Participants saw this strategic change in implementation as a long-term outcome of the training. Some organizations shifted their approach to evaluation after participating in the training to be more oriented toward demonstrating successful outcomes and not overburdening staff and clients with unusable forms. This included improvements in evaluation planning, understanding of data collection, and use of collected data for further improvement.

## Discussion

Introducing, implementing, and sustaining the use of effective policies, programs, and practices is a key component of professional practice for leaders in the fields of behavioral health, public health, and medicine. This innovative and well-received training program addresses a widely recognized gap in implementation research and practice. Although a growing number of programs provide training in dissemination and implementation research, few programs accept or are designed for those engaged in implementation practice. This training responds to the identified need of training individuals in implementation roles, such as administrators, supervisors, practice improvement facilitators, and frontline providers [3].

The mixed-method results provide a number of complimentary insights into the perceived value of the training and its ability to support organizational leadership in their ability to implement evidence-based practices. Foremost, both the quantitative and qualitative results indicate that participants reacted positively to the training, felt that it was appropriate, and made many positive comments about the training when interviewed. As others have noted, the techniques or strategies used to promote the integration of evidence into practice is critical. Our findings suggest that a short training such as TRIPLE at a minimum is an acceptable and appropriate means for educating organizational leadership about implementation practice.

Results also indicate that TRIPLE shows promise in its ability to increase participant knowledge of implementing evidence-based practices *and* change participant behavior related to implementing evidence-based practices. After completing TRIPLE, participants reported greater degrees of perceived self-competence and more resilient response to critical issues of implementation even in the face of inevitable challenges. Furthermore, the qualitative results suggest that participants were able to enact behavior change in a number of ways, for example engaging additional staff and other stakeholders when implementing a new practice.

Lastly, our evaluation indicated that participants were able to stimulate small changes within their organizations. Participants reported that implementation climate improved following the training program although none of the participants were able to implement a practice in the time frame of our evaluation. Because implementing a new practice often takes significant effort and planning over several months or even years, it was not our expectation that we would be able to detect change in the services organizations provide. However, TRIPLE showed promise in its ability to improve organizational-level factors known to influence implementation success, [12, 13] for example, being intentional about collecting information for monitoring and evaluation purposes. The reported changes in implementation climate particularly signal enhanced efforts to support, reward, and value expertise in EBP implementation following participation in TRIPLE.

We acknowledge several limitations in the training and evaluation. Because the CDI heavily subsidized program costs, we cannot assess the marketability and sustainability of TRIPLE. While agencies were willing to pay the $200 fee, we cannot assume that full training costs could be supported by tuition. Consistent with its service mission, the university supported on an “in kind” basis the time investment of all but one trainer, whose travel and time were paid on a consultant basis. The evaluation results should be viewed as exploratory. This was a small-scale pilot project conducted with leadership in substance abuse organizations. We did not include a control group to which we could compare outcomes. Although the training content was drawn from health services, we cannot claim that TRIPLE would be equally valuable in other public health or social service areas. The sample size for quantitative analyses was small, yet we used paired samples *t* tests that are particularly robust with small samples [17]. Our post hoc power calculations indicate we were sufficiently powered for the ICS and ILS, but not the ORIC, which may have contributed to the null ORIC findings. Our qualitative analyses are also limited in their generalizability. However, mixed-methods evaluations are widely recognized as robust research and evaluation designs, particularly with small sample sizes, because various modes of data collection provide the opportunity to triangulate outcomes on similar concepts [24]. In our case, we found that the quantitative and qualitative results were highly complementary to each other.

## Conclusions

Our primary findings are threefold: (1) practice leaders were highly receptive to TRIPLE, reporting it to be both acceptable and appropriate for their training needs; (2) TRIPLE appeared to improve practice leaders’ knowledge and behaviors related to evidence-based interventions; and (3) although it was not feasible to demonstrate more successful EBP implementation within organizations given constraints of time and resources, TRIPLE did improve organizational-level factors known to influence implementation success. There is both need and demand for implementation practice training. TRIPLE may help fulfill a critical need for emerging behavioral healthcare leaders and their organizations. The program aims to enhance translatable leadership skills of emerging leaders who are responsible for the high-quality delivery of a wide range of EBPs. Implementation research provides frameworks, evidence about implementation strategy appropriateness and effectiveness, and methods for assessing implementation outcomes; these skills can be leveraged to better equip organizational leaders for their roles as implementers and implementation champions. Indeed, such lessons from implementation science conveyed through leadership training may have more direct and greater influence on practice change than passive dissemination of results through professional publications alone. With relatively modest burden on faculty and research staff, academic settings can provide short-term training to better equip their affiliated clinical sites for implementing practice improvements.

### Additional files

Additional file 1: TRIPLE Invitation Letter, as sent to Agency Directors and CEOs. (DOCX 23 kb)

Additional file 2: Semi-Structured Interview Questionnaire. (DOCX 18 kb)

## Data Availability

The datasets used and/or analyzed during the current study are available from the corresponding author on reasonable request.

## Appendix

**Table:** Qualitative codebook

Main categorical code	Thematic code	Description	Example
Reaction—overall perception of the course	Positive receptivity to the training.	Reactions to the opportunity to participate in the training.	
	Positive about training	Positive comments about the course, the materials, and the structure of the course. Can also include comments about things that were done well, and not necessarily be overtly positive.	“I really enjoyed it.” “I liked the time frame.” “You were pretty thorough about the getting out the information.”
	Negative/or suggested change	Negative comments about the course, the material, and the structure of the course. Can also include comments about things that could be done differently or improved.	“I did not always know, or felt like it was completely applicable to the work we do here.”
Learning—knowledge gained; attitude and belief changes; change in awareness.		Knowledge gained, changed perceptions about EBP, or more consideration of EBPs as part of service delivery. Changes in thinking specific to the individual.	“My knowledge based was certainly improved.” “I think I’m more aware of it. For example..” “It made me more mindful of buy-in.”
Behavior—changes in how the participants behave in their job role		Changes within the participant in communication style, efforts to improve buy-in, planning, strategizing, etc.	“It helped me to think more deeply…and to plan for some of the barriers and some of the things that get in the way of successfully implementing a practice.” “As I was training people, there were some people that did not really this it was a good idea…it has changed how I approach some people in training, to work more not so much on the ins and outs, but why we are doing it.” “Having more intentional conversations.”
Results—changes at the agency level	Changes in service delivery or practice.	Attempting to implement or successfully implementing new EBPs. Considering the evidence-base for current practices and making adjustments to achieve fidelity.	“We’re looking a little more critically at some of the stuff that we implement…” “Revamping what is already existing.”
	Changes in agency culture or climate regarding EBP	Changes in how other administrative staff view EBP or implement services. Enhanced collaboration among staff.	“I think there is more buy-in.” “We all just have a heightened awareness of how we are approaching and how we are implementing things rather than just jumping in.”
	Improved staff knowledge/training	Changes made to help frontline staff learn more EBPs, or understand why the agency uses EBPs	“Helped new staff get training in different evidence-based practices starting at hiring has been improved.” “We have been spending more time on different tips and techniques in our weekly staff meetings.”
	Changes in evaluative approaches	Changes in data collection forms and how evaluation is approaches within the agency.	“we have streamlined our registration forms.” “The way that we evaluated or programs, and look at our data, in terms of outcomes and what is the best things for our clients.”
	Other	All other changes that do not fit into the other categories.	
Barriers		Factors that inhibited, slowed, or made change difficult either at the participant or organizational level.	“I was a little frustrated by the (lack of) resources for client strategies.” “There is just so many moving parts that sometimes making a change…is a hard thing to do….”

## Abbreviations

CAPA: Community Academic Partnership on Addiction
CDI: Center for Dissemination and Implementation
CEO: Chief Executive Officer
EBP: Evidence-based practice
ICS: Implementation Climate Scale
ILS: Implementation Leadership Scale
IPH: Institute for Public Health
ORIC: Organizational Readiness for Implementing Change Scale
SD: Standard deviation
TAAS: Training Acceptability and Appropriateness Scale
TRIPLE: Training in Implementation Practice Leadership
