# Supplementary material for: Training in Implementation Practice Leadership (TRIPLE): evaluation of a novel practice change strategy in behavioral health organizations
Source: Implement Sci. 2019 Jun 20;14:66. doi: 10.1186/s13012-019-0906-2 (PMC6585005; doi:10.1186/s13012-019-0906-2)
Supplement: Supplementary file 1 — TRIPLE Invitation Letter, as sent to Agency Directors and CEOs. (DOCX 23 kb) [file 13012_2019_906_MOESM1_ESM.docx]

**Appendix A: TRIPLE Invitation Letter, as sent to Agency Directors and CEOs**

**Community Academic Partnership on Addiction Training in Implementation Practice Leadership (CAPA-TRIPLE)**

Dear ___________:

I write to share information about and invite your application to an exciting training opportunity, a benefit currently available exclusively to members of the [Community Academic Partnership on Addiction (CAPA)](mailto:https://addiction-partnership.wustl.edu/about-us/). We are inviting emerging leaders of behavioral health programs who were nominated by their agency’s leadership to participate in our CAPA Training in Implementation Practice Leadership from 8:00AM to noon on the following three Fridays:

January 27^th^ 2017
February 17^th^ 2017
March 3^rd^ 2017

In addition to half-day in-person sessions on these dates, this training program will include interim support (in between sessions) and periodic follow-up support from training faculty and staff. You have been nominated for this training by your agency’s leadership as someone who shows high potential to be a future leader of programs and organizations in behavioral health.

Benefits of participation in this training program include the following:

- Leadership skill building for implementing and evaluating evidence-based practices
- Access to expert faculty in behavioral health implementation practice
- Published literature and hands-on training exercises
- Up to 3 months of follow-up support (following completion of the sessions)
- Interaction and collaboration with peers from within and outside your agency
- CEUs (10 credits)
- Certificate of completion of training
- Participation at the significantly subsidized cost of $200, which includes a textbook and parking

Selection to the CAPA Training in Implementation Practice Leadership Program requires a commitment to actively participate in three in-person sessions (4 hours each), engagement in recommended readings and exercises before sessions (approximately 2 hours per session), and a willingness (of the participant and the participant’s agency) to trial small-scale changes within participants’ respective agencies to develop skills in leading implementation practice. The change may be one already underway or a new change your agency is planning. We have requested that agency CEOs participate in the first hour of the first session (January 27^th^).

This training program offers a tremendous opportunity to develop leadership skills and increase competency at effectively implementing and evaluating evidence-based practice among members of your team at very little cost to your organization.

We would like to invite you to apply by completing the attached form and emailing it to [DandI@wustl.edu](mailto:DandI@wustl.edu) by January 17^th^. We have the capacity to select one to three participants from each organization.

Sincerely,


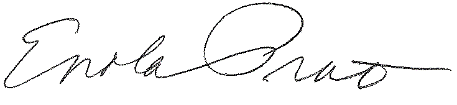


Enola Proctor
