# Supplementary material for: Training in Implementation Practice Leadership (TRIPLE): evaluation of a novel practice change strategy in behavioral health organizations
Source: Implement Sci. 2019 Jun 20;14:66. doi: 10.1186/s13012-019-0906-2 (PMC6585005; doi:10.1186/s13012-019-0906-2)
Supplement: Supplementary file 2 — Semi-Structured Interview Questionnaire. (DOCX 18 kb) [file 13012_2019_906_MOESM2_ESM.docx]

**Appendix B: Semi-Structured Interview Questionnaire**

Introduction: Hi this is [your name]________ from the Center for Dissemination and Implementation. We scheduled an interview with you about your participation in the TRIPLE training. Is now still a good time? I work with Enola and Alex. The purpose of the interview today is to follow up with you about the TRIPLE training and see how things have been going for you at [name of agency]_______________. We also would like to get your opinion about how the training could be improved or any other feedback that you might have. The interview should take about 25 minutes, and just as a reminder you’re free to skip any questions or stop the interview at any time. Do you have any questions for me before we get started?

Note: we will send a version of the interview questions to participants ahead of time by email. Also, if using prompts for a question restate the question after the prompts.

**Change in proximal factors – 10 min**

1. Let’s go back to when you learned about this, can you tell me very briefly what led you to participate in this training?
2. What, if anything, changed with your professional capacity or style because of the training program?

**If they’re having trouble responding:** *These could be things such as:*

- 1. Leadership style?
  2. Effectiveness within your team/agency?
  3. Knowledge?

1. What changes, if any, have taken place with your team/agency because of the training program?

**If they’re having trouble responding:** *These could be things such as:*

- 1. *How your organization approaches implementation?*
  2. *Addresses quality improvement?*
  3. *Assesses cost?*
  4. *New meetings or workgroups?*
  5. *New resources gathered (e.g., websites, tools, checklists)?*

**EBP implementation – 10 min**

To get ready for talking with you I reviewed your workbook. I understand that you selected [EBP]_________ as the intervention/practice that you wanted to *implement/improve the implementation of* in your agency, is that correct?

1. In thinking about this evidence-based intervention, first tell me a little about what you had hoped to accomplish?

**If they’re having trouble responding:**

- 1. Were you trying to newly adopt/improve/adapt/de-implement this intervention?
  2. Improve the quality or adapt it in some other way?
  3. Reduce or discontinue its use?

1. Would you say that your agency was able to [adopt/improve/adapt/discontinue] the practice/intervention?

**[If yes/successful]**

1. In what ways, if any, do you think the training program led to changes in how you are implementing this intervention?
2. Can you describe for me what steps you’ve taken toward implementing/discontinuing the practice/ intervention?
3. As you may recall, part of the training was an opportunity to reflect on what strategies your agency has used to facilitate or enhance your implementation efforts since the training. These could include additional training experiences, use of educational materials, financing changes, restructuring efforts, quality improvement approaches, or even policy changes. What strategies, if any, has your agency used to bring about improvements in implementation since the training?

**[if not successful]:**
That’s ok. We know that there is a wide range of experiences and that success in implementation is an ongoing process. We just want to understand what happened so that we can better support people in the future.

1. Were there things from the training that you tried, but they didn’t work?
2. Can you describe any other barriers or frustrations you experienced?

**[for everyone]:**

1. Can you describe any other significant changes that may have taken place in the agency that you think had an influence on what happened?
2. Can you describe any unanticipated changes, positive or negative, that may have taken place in your agency as a result of the training?

**Feedback about training – 5 min.**

These last few questions are about the training itself and what you think we could do better.

1. As you may recall, we invited about two clinical leaders from each agency to participate in this training. Looking back on the training, do you think that was the right group for us to target?
   1. What would be the best target audience for this training?
2. What aspects of the training were most useful for you?

**If they’re having trouble responding:** *These could be things such as:*

- 1. *Resources provided*
  2. *Group sessions*
  3. *Work sheets*
  4. *Teleconferencing*
  5. *Information provided*

1. What aspects of the training were not useful for you?

**If they’re having trouble responding:** *These could be things such as:*

- 1. Resources provided
  2. Group sessions
  3. Work sheets
  4. Teleconferencing
  5. Information provided

1. What changes do you think we should consider making to improve our training program in the future?
2. Would you suggest any changes in the format of the training (e.g., number of sessions, length of sessions, time between sessions, etc.)?
3. What other comments or suggestions do you have regarding the training program?

**Wrap up**

1. If I have any follow up questions, is it ok for me to send you an email?

Thanks again for talking with me. *Anything else that you think should be said to wrap things up.*
